# Experimental Systems for Measuring HIV Latency and Reactivation

**DOI:** 10.3390/v12111279

**Published:** 2020-11-09

**Authors:** Koh Fujinaga, Daniele C. Cary

**Affiliations:** 1 Division of Rheumatology, Department of Medicine, School of Medicine, University of California, San Francisco, CA 94143-0703, USA; 2 Department of Medicine, Microbiology, and Immunology, School of Medicine, University of California, San Francisco, CA 94143-0703, USA; carydc@hotmail.com

**Keywords:** HIV, latency, latency reversing agents, transcription, P-TEFb, silencing, transcription interference, NFkB

## Abstract

The final obstacle to achieving a cure to HIV/AIDS is the presence of latent HIV reservoirs scattered throughout the body. Although antiretroviral therapy maintains plasma viral loads below the levels of detection, upon cessation of therapy, the latent reservoir immediately produces infectious progeny viruses. This results in elevated plasma viremia, which leads to clinical progression to AIDS. Thus, if a HIV cure is ever to become a reality, it will be necessary to target and eliminate the latent reservoir. To this end, tremendous effort has been dedicated to locate the viral reservoir, understand the mechanisms contributing to latency, find optimal methods to reactivate HIV, and specifically kill latently infected cells. Although we have not yet identified a therapeutic approach to completely eliminate HIV from patients, these efforts have provided many technological breakthroughs in understanding the underlying mechanisms that regulate HIV latency and reactivation in vitro. In this review, we summarize and compare experimental systems which are frequently used to study HIV latency. While none of these models are a perfect proxy for the complex systems at work in HIV+ patients, each aim to replicate HIV latency in vitro.

## 1. Introduction

Antiretroviral therapy (ART) regimens keep the virus below the level of detection in HIV+ individuals. However, upon cessation of therapy, plasma viremia rapidly rebounds, leading to the progression to AIDS [1]. Although ART is successful at suppressing HIV, it is unable to completely eradicate the virus due to the presence of latent HIV reservoirs [2]. These reservoirs are long-lived viral sanctuaries scattered throughout the body. In HIV+ individuals, HIV-infected cells can be detected not only in the peripheral blood and lymph nodes, but also in the brain, lungs, kidneys, liver, adipose tissues, gastrointestinal tract, genitourinary systems, and bone marrow. Lymphoid tissues, such as spleen, thymus, and particularly gut-associated lymphoid tissues (GALT), are the most important viral reservoirs, which remain relatively dormant, but account for the immediate rebounds in viremia following cessation of ART [1,3]. One currently investigated approach to target the latent reservoir is therapeutic reactivation of HIV, also known as “shock and kill” [4,5]. A latency reversing agent (LRA) or combinations of LRAs would be used to reactivate and shock the virus out of hiding, leaving it vulnerable to immune killing machinery or cytolysis by the virus [4,5,6,7]. However, sufficient levels of HIV reactivation will be required for “shock and kill” therapies to significantly impact the reservoirs. Therefore, to develop the most effective reactivation strategies, it is essential to understand the precise cellular and molecular mechanisms by which HIV latency is established and controlled [8].

There are no animal models which fully recapitulate HIV pathogenesis as observed in HIV+ individuals [9]. Many studies on HIV latency and reactivation instead rely on tissue culture systems using immortalized human cell lines or primary cells derived from human blood or tissues [10,11]. These in vitro culture models utilize different combinations of growth factors, feeder cells, and cytokines to drive HIV-infected, activated T cells into a quiescent state, with the goal of modeling the conditions of latently infected T central or effector memory (Tcm or Tem) cells [12,13,14]. While all of these models produce resting T cells with transcriptionally silent HIV, it is unclear whether any of these in vitro latent HIV models phenocopy latent reservoirs in HIV+ individuals. In the following review, we present several stable cell line and primary cell models used to study HIV latency in order to provide a comprehensive comparison of the different available systems.

## 2. Mechanisms Regulating HIV Transcription and Latency

**Figure 1:**
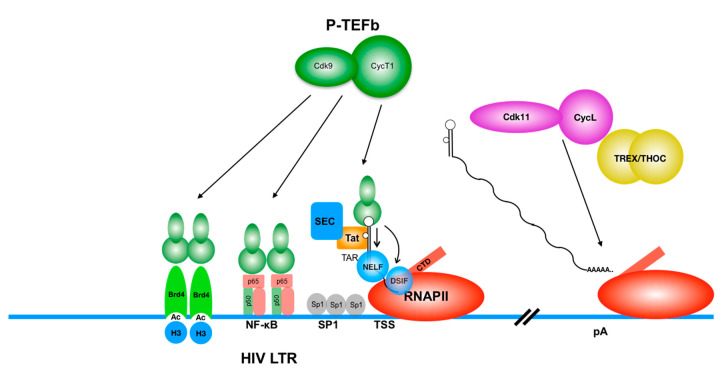
Two major cellular cyclin/Cdk complexes play key roles in HIV transcription. P-TEFb (CycT1:CDK9, green) is recruited to the transcription machinery on HIV LTR by various factors. Epigenetic factor Brd4, DNA-bound transactivator NFκB, and viral Tat (associated with cellular super elongation complex) bind to CycT1 and recruit P-TEFb to HIV provirus in an active chromatin environment. P-TEFb then phophorylates RNAPII and negative transcription elongation factors NELF and DSIF, which augments transcriptional elongation. CycL:Cdk11 complex (yellow), in turn, associated with transcription/export and THO complex (TREX/THOC) is recruited to RNAPII transcribing HIV genes. Cdk11 phosphorylates the CTD of RNAPII and promotes the assembly of cleavage and polyadenylation (CPA) factors at the 3′ end of genes, which ensures optimal expression of HIV mRNAs.

HIV transcription is initiated at the transcription start site (TSS) located in the HIV long terminal repeat (LTR) region, which consists of a typical inducible promoter/enhancer sequences [15] (Figure 1). Once integrated, the HIV LTR acts like a typical inducible cellular gene promoter, responding to various cellular signaling cues [15]. HIV transcription is regulated by host transcription factors (TFs) which control host gene expression. Following infection, activated cells can return to a resting state. These quiescent cells have vanishing low levels of important TFs. This maintains viral latency by keeping these cells in a transcriptionally silent state. Multiple cellular signaling pathways can control HIV transcription positively: positive transcription elongation factor b (P-TEFb), the nuclear factor kappa B (NFκB) pathway [16], the nuclear factor of activated T cells (NFAT) pathway [17], the mitogen-activated protein kinase (MAPK) pathway [18], the toll-like receptor (TLR) pathway [19], and the mammalian target of rapamycin (mTOR) pathway [20]); as well as negatively: via recruitment of the polycomb repressive complex (PRC) [21,22,23], the Cullin 3 E3 ligase pathway [24], and the zinc-finger protein 304 [25]. The proviral 5′-LTR is an inducible promoter containing a TATA-box and upstream enhancer binding regions [26]. For example, a typical HIV LTR contains two (clade B) or three (clade C) NFκB sites and two SP1 sites [26].

Immediately after initiation of transcription and promoter clearance, the host RNA polymerase II (RNAPII) is stalled by the negative elongation factor (NELF) and DRB-sensitivity inducing factor (DSIF), resulting in accumulation of short premature RNAs with a length of less than 100 nucleotides that form a stem-loop structure called the transactivation response (TAR) element [27]. When there is sufficient HIV Tat, it binds to TAR and the cyclin T1 (CycT1) subunit of P-TEFb. The cyclin-dependent kinase 9 (CDK9) subunit of P-TEFb then hyperphosphorylates the C-terminal domain (CTD) of RNAPII at the second serine residues (S2) of the heptapeptide repeats (YSPTSPS), NELF and the SPT5 subunit of DSIF. NELF is then removed, and DSIF becomes a positive transcription factor, which accompanies RNAPII as it progresses through the gene body [28,29,30,31]. Recruitment of P-TEFb is a main switch between non-productive and productive transcription of HIV. Importantly, in quiescent CD4+ T cells, such as memory T cells [32,33] and hematopoietc stem cells [34,35,36], which represent the bulk of the latent reservoir, the expression of CycT1 is severely downregulated post-transcriptionally [37,38]. This downregulation of CycT1 causes a block of transcription elongation from the HIV LTR.

In the late stage of HIV transcription, another cellular CDK/cyclin complex (CDK11/CycL) is recruited to RNAII elongation complex transcribing HIV [39,40]. CDK11 phosphorylates RNAPII’s S2 and recruits the transcription export (TREX/THO) complex, which promotes the cleavage and polyadenylation of HIV transcripts, stabilizing HIV mRNAs and enhancing their nuclear export and translation of viral proteins [39]. Interestingly, CDK11/CycL expression is also severely downregulated in quiescent CD4+ T cells [41]. Therefore, at least two major cellular CDK/cyclin complexes play key roles in HIV latency [42] (Figure 1). Recent studies from Yukl and colleagues demonstrated that HIV transcription is blocked at multiple steps including initiation, elongation, splicing, termination, and polyadenylation [43].

**Figure 2:**
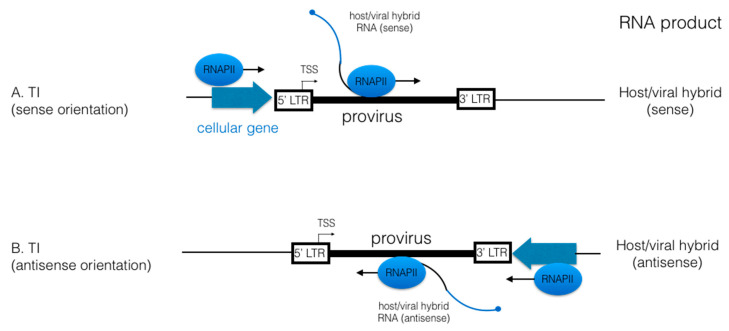
Host–viral hybrid RNAs are expressed by TI in latently infected cells. (**A**) When HIV integrates in the sense orientation within a host cell gene, the transcribing RNAPII reads through the boundary between the host gene and the 5’LTR or the HIV proviral DNA, and terminates in the 5′-LTR (past TAR near the poly A site), resulting in the expression of host–viral hybrid (HVH) RNAs. (**B**) When HIV integrates in the antisense orientation within a host gene, RNAPII reads through without stopping and HVH RNAs containing HIV antisense RNA are produced.

HIV proviruses are integrated into host chromosomal DNA at random locations. There is a preference to integrate inside actively transcribing genes [44,45], which have less compact chromatin structure. Thus, HIV integration site is a key factor to understanding how viral latency is established and maintained. When HIV is integrated into a transcriptionally active gene, HIV transcription from its 5′-LTR is silenced by a mechanism called transcription interference (TI) (Figure 2). RNAPII actively transcribing cellular genes reads through the boundary between host gene and HIV provirus, preventing viral transcription initiated at the TSS in the 5′-LTR, producing host–viral hybrid (HVH) RNA [46,47], although a certain level of HIV transcription occurs at the TSS when HIV is integrated in the same orientation as host genes [47]. Since HIV provirus can be integrated in the same or opposite direction to the cellular gene, HVH can contain sense or antisense (AS) HIV RNA. These RNAs are not transcribed from HIV’s canonical TSS and therefore they do not produce HIV proteins. To reverse TI and increase HIV mRNAs, it is necessary to block the upstream cellular transcription and stimulated HIV transcription from its canonical TSS at the same time [46].

It is also plausible that HIV transcription is regulated epigenetically via histone modifications, in a manner similar to host genes [11,48,49,50,51,52]. Early seminal studies by Verdin and colleagues demonstrated that a highly ordered nucleosome structure, called Nuc 1, is built into the region of HIV TSS. Chromatin remodeling of Nuc1 is associated with HIV latency and reactivation [53]. While the role of DNA methylation on HIV latency is still debatable [54], histone modifications play critical roles in HIV latency and reactivation [55]. In particular, repressive histone marks such as H3K9 methylation and H3K27 methylation can be found at the integrated HIV LTR in latently infected cells. H3K27 methylation is mediated by the PRC via direct recruitment of the EZH histone methyltransferase (HMT) subunit to HIV LTR [22,56]. A selective EZH2 inhibitor exhibited synergistic reactivation of HIV from latently infected cells when used in combination with other LRAs [56]. H3K9 methylation on the HIV LTR occurs via various HMTs: SUV39H, G9a, and/or SETDB1 [57,58]. It has recently been suggested that crosstalk between H3K9 and H3K27 methylation might occur at the HIV LTR, driving HIV into a deeper latency [59,60]. On the other hand, transcriptionally active histone marks such as H3K4 and H3K27 acetylation can be found upon HIV reactivation [55]. In latently infected cells, these lysine residues are deacetylated by various histone deacetylases (HDAC) recruited to the HIV LTR [61,62]. HDAC inhibitors such as vorinostat were among the first compounds used in clinical trials for HIV latency reversal and functional cure [63]. Additionally, a recently discovered histone modification, crotonylation, may be involved in HIV latency [64]. Induction of histone crotonylation by the crotonyl-CoA-producing enzyme acyl-CoA synthetase short-chain family member 2 (ACSS2) reactivates HIV from latently infected cells [65].

## 3. Viral Expression in Latently Infected Cells

Although latently infected cells are not actively replicating, and do not express detectable viral antigens, these cells produce several different viral RNAs and/or proteins [43,66]. In particular, the majority of HIV proviruses detected in HIV+ individuals are defective and unable to produce infectious viruses because of mutations caused by cellular RNA-editing enzymes such as APOBEC 3G [66,67,68,69]. Cells bearing these defective proviruses can still produce viral proteins and RNAs [66,67]. Short viral transcripts, formed through the block at transcription elongation, were among the first viral RNAs detected in peripheral blood mononuclear cells (PBMCs) from asymptomatic HIV-infected individuals [43,70,71,72]. With the development of highly sensitive and specific quantitative reverse transcription PCR (RT-qPCR) and high throughput RNA-sequencing (HT-RNAseq), different viral RNA species, including multiply and singly spliced and unspliced HIV mRNAs, as well as nonproductive HVH RNA resulting from TI, are able to be detected [43,46,47,73]. Analysis of which viral RNAs are produced by latent cells, either while still at rest or upon reactivation, can be used to further understand HIV latency. The HIV LTR can also produce antisense (AS) transcripts, as is the case of most of cellular promoters [74,75,76,77]. However, it is still unclear whether the levels of AS HIV transcripts correlate with HIV latency. These AS HIV transcripts could encode proteins, but it is unclear if these proteins are expressed in latently infected cells [78]. The ratio of sense to AS transcripts produced by a reactivated cell can inform whether a strategy is successful in truly reactivating the latent virus. Some HIV RNAs expressed in latently infected cells can be exported as extracellular vesicles, in either exosomes or virus-like particles. Exosomes containing HIV components such as Nef proteins or TAR RNA are detected in HIV-infected individuals [79,80,81,82,83,84,85,86]. Exosomes in patient sera or cell supernatants can be isolated and analyzed for the presence of Nef or TAR to further understand latency. The majority of HIV-infected cells contain defective HIV proviruses, and a very low population of HIV-infected cells retain the ability to produce infectious progeny viruses [87]. Defective HIV proviruses often contain a large deletion in the LTR and/or coding regions [88,89,90]. It is becoming clear that some model systems overestimate the reactivatable reservoir, by being unable to distinguish between defective and infective clones [91].

## 4. LRAs and How They Work

To increase HIV transcription from latently infected cells, it is necessary to manipulate cellular pathways that are involved in the establishment and maintenance of HIV latency. However, optimal HIV reactivation can only be achieved by stimulating HIV transcription to produce enough Tat to boost HIV transcription with the aid of the cellular P-TEFb [15,27,92,93]. Initial rounds of HIV transcription must occur without Tat, which is a very inefficient process. Once sufficient Tat is produced, viral transcription is amplified hundreds to thousands of times. Thus, this self-fueling feedback mechanism regulated by Tat is the primary “on/off” molecular switch between latent and productive infection of HIV [15,27,92,93].

Most LRAs aim to increase initial HIV transcription [19,94]. To date, there are several categories of LRAs being tested preclinically and clinically: (1) P-TEFb releaser/activators [42], (2) protein kinase c (PKC) agonist/canonical NFκB inducers [95], (3) non-canonical NFκB inducers, (4) Toll-like receptor agonists [19], (5) epigenetic modulators [96], (6) mTOR inhibitors [20], (7) protease inhibitors (PIs), (8) MAPK agonists [97], etc. Examples of LRAs are presented in Table 1. These LRAs were identified and tested with various latency models including cell lines, primary cells derived from HIV-seronegative individuals infected with HIV reporter virus ex vivo, or primary cells derived from HIV+ individuals. However, since mechanisms of HIV latency vary depending on cell types or even cell clones, none of these LRAs optimally reactivate HIV in all latency models.

Cellular factors and cellular pathways mediating HIV latency and reactivation also vary among cell types. It is still largely unclear exactly where HIV-infected cells reside and how HIV latency is established and maintained in HIV+ individuals in vivo. Because of the complexities of viral latency and insufficient knowledge about the viral latency in vivo, many compounds that reactivate HIV transcription in cell line systems do not reverse latency in HIV+ individuals [128,129]. Therefore, it is necessary to understand mechanisms of HIV latency in different models to identify effective LRAs that can be used in the HIV anti-latency therapies (HALT) and to choose appropriate latency models to screen new compounds. In the following sections, we will discuss the differences between different systems that model HIV latency.

## 5. HIV Molecular Clones

Infectious molecular clones are an important tool of modern virology, which can be used to produce infectious viral particles and enable genetic analysis of virally encoded genes [130,131,132]. For HIV, several infectious molecular clones were constructed immediately after the discovery of the virus. Since HIV can infect non-dividing cells, molecular clones were further modified to develop safe and efficient lentiviral delivery for non-HIV gene therapies including treatment for cancer and genetic disorders such as Wiskott-Aldrich Syndrome and metachromatic leukodystrophy [133,134,135,136,137]. To prevent spreading infection, lentiviral vectors do not contain viral structural and regulatory genes which are essential for viral replication, such as *gag*, *pol*, *env*, which can be supplemented by different plasmids [138]. These lentiviruses do not produce progeny viruses after infection, hence they do not replicate, resulting in a single round of infection. The envelope protein of Vesicular Stomatitis Virus (VSV-G) is often packaged with the replication incompetent HIV in order to produce virus particles which can enter into the host cells independent of the HIV envelope protein (Env) [138]. Lentiviral vectors encoding reporter genes including cDNAs for fluorescent proteins (FPs) and luciferase (Luc) have been constructed to study aspects of HIV infection in vitro. In particular, EGFP and mCherry are useful tools to measure viral gene expression and viral gene integration independently [139,140,141,142]. The gene expressing the fluorescent protein can be placed after the HIV promoter to measure HIV transcription, or from a separate promoter to measure expression of the gene in which HIV has been integrated [139,140,141,142].

## 6. Choosing the Best Latency Models

The choice of appropriate models to study HIV latency and reactivation depends on the objectives of the study. Since HIV only infects and causes AIDS in humans, there are no animal models that precisely recapitulate the course of HIV infection. Among a few exceptions are primates infected with an HIV-simian immunodeficiency virus (SIV) hybrid virus (SHIV) and severe combined immunodeficiency (SCID) mice transplanted with human hematolymphoid tissues (SCID-hu mice) infected with HIV (since animal models are beyond the scope of this review, please refer to other reviews for further reading) [143,144,145,146].

For cell culture systems, a major question is whether to use primary cells or cell lines. Since HIV infects CD4+ T cells and macrophages, primary CD4+ T cells and macrophages from PBMCs or lymphoid tissues are the experimental systems closest to HIV infection in humans [14,147]. However, isolating and culturing these cells from biopsy samples are generally cumbersome processes that require experience and skill [148]. These cells can be kept ex vivo for only a certain length of time, usually 2–4 weeks, although it is possible to prolong these cells by ectopically expressing anti-apoptotic factors [149,150]. Moreover, the efficiency of transfection or electroporation of exogenous genes or proteins into these cells are generally low. Sample-to-sample variations can be large and it might require preparation of a large number of samples to obtain statistically reliable data.

On the other hand, immortalized cell lines are easy to maintain for a long time with higher transfection efficiencies. Popular cell lines used in HIV researches are Jurkat, CEM, MOLT4, MT 4 for T cells, and THP1 and U937 for monocyte/macrophages [11]. However, since cell lines are actively replicating, the cellular characteristics are different from hematopoietic cells in humans. Of particular importance, the level of transcription machinery, namely P-TEFb, is severely downregulated in primary resting CD4+ T cells and macrophages whereas these factors are highly expressed in cell lines [15,41,42]. Other cellular transcription machinery components and/or transcription factors are similarly downregulated in quiescent cells, resulting in blockages of multiple steps of HIV transcription [43]. HIV transcription seems to be blocked at different steps between circulating cells (blood) and lymphoid-associated cells (GALT, for example) based on RNA expression analysis [151,152]. On the other hand, because of the nature of active cell replication, global transcription is kept at relatively high level in cell line models. Regulation of HIV latency occurs largely at the step of transcription elongation [153], due to impaired recruitment of Tat/P-TEFb complex to HIV TAR via transcription interference or mutations in Tat and/or TAR [153]. This may affect cellular responses to LRAs in cell lines compared to primary cell systems. In the following section, we will further discuss several HIV latency models.

## 7. Primary Cells (PBMCs) from HIV+ Individuals vs. Ex Vivo Infection Models

Primary CD4+ T cells and macrophages can be obtained from blood samples or tissue biopsies from HIV+ or HIV-seronegative individuals. Cells from HIV+ individuals contain naturally occurring latently infected cells. However, to obtain and use HIV+ primary cells requires special safety precautions and facilities, as well as lengthy administrative process. The proportion of latently HIV-infected cells in primary cells is estimated to be 1 in million or less. Therefore, it is very difficult to detect HIV-expressing cells and measure HIV reactivation at the single-cell level. On the other hand, de novo infection with HIV reporter virus of primary cells from HIV-seronegative individuals generally provides a larger proportion of HIV latently infected cells and therefore is an appropriate model to study viral latency and reactivation at the molecular/single cell level. In general, quiescent CD4+ T cells are isolated from PBMCs or lymphoid tissues such as tonsils or the gut [147,148,154], and infected with pseudotyped single-round HIV encoding FPs or other reporters. When a dual-color HIV reporter virus such as Duo-Fluo [139,140] or RGH [141,142] is used, a bulk of HIV-infected cells are sorted by the internal promoter-driven mCherry expression, then latently infected cells are negatively selected by HIV-driven EGFP expression. These cells are further used to study the mechanisms by which HIV latency is established and maintained, and to measure responses to different LRAs. Although lentiviral transduction normally requires cell stimulation, direct infection of resting CD4+T can be achieved, with less than 1% efficiency, by mixing Tcm or Tem with a high titer of HIV (m.o.i ~22–150) [155]. Recently, Greene and colleagues developed an efficient method to transduce a lentiviral vector into quiescent cells by using a helper virus containing SIV’s Vpx (Vpx-VLP) [156].

There are several different well-established protocols of latent cell isolation and HIV infection established by various laboratories. In particular, the following methods were designed to obtain latently infected cells without T cell stimulation/re-entry to latency. Karn and colleagues demonstrated that a high number of latently infected cells are obtained by co-culturing HIV-infected CD4+ T cells and H80 feeder cells [157]. The method established by Bosque and Planelles uses TCR stimulation in the presence of TGF-β and anti-IL4/anti-IL2 to differentiate CD4+ T cells into non-polarized subsets, representing Tcm [158]. In the Siliciano method, cells are first set to a resting state in the presence of anti-apoptotic factor Bcl2 to prolong the culture, then cells are infected with HIV and latently infected cells are sorted [159]. Spina and colleagues found that HIV infection can be established by directly infecting freshly isolated resting CD4+ T cells [14]. The method developed by Greene and colleagues also uses direct HIV infection of resting CD4+ T cells with spinoculation in order to increase the interaction between HIV and CD4+ T cells [160]. The model established in the Lewin lab uses chemokines such as CCR7, CXCR3 or CCR6 to increase the efficiency of HIV infection in resting CD4+ T cells [161,162]. While no one model perfectly replicates latently infected cells, each of these model systems attempts to recapitulate the in vivo conditions to create a cell system that will permit the latently infected cells to be manipulated in culture. Planelles and colleagues compared the responses of different classes of LRAs known to reactivate HIV in latently infected cells on different primary latent model cells, and demonstrated that none of the tested models accurately capture the response characteristics of latently infected cells from HIV+ individuals [14]. Chomont and colleagues recently demonstrated that LRAs such as HDACis and PKC agonists display variable activities in different subsets of primary memory CD4+ T cells, suggesting that combination of several compounds might be required for an optimal latency reversal in all memory subset [33].

## 8. Measuring HIV Transcription/Gene Expression in Primary Cells Derived HIV-Positive Individuals

While HIV gene expression can be measured by various sensitive methods in ex vivo infection models using pseudotyped reporter HIV, there are limited number of methods available for measuring viral gene expression in latently infected cells derived from HIV+ individuals. The most sensitive assay is to directly measure HIV mRNAs is using RT-qPCR. By using different sets of PCR primers, different HIV mRNA species (unspliced, singly or doubly spliced, AS, HVH, etc) can be measured independently. However, some HIV species (particularly, short premature/unproductive RNAs) may be already highly expressed in latently infected cells. Therefore, primers specific for the *gag* genes, to detect unspliced HIV RNA, are often used to measure viral reactivation [163]. Recently, a new PCR technology called droplet digital PCR (ddPCR) was developed and applied to measure HIV RNAs [43,72]. In this system, a sample is separated into tens of thousands of droplets of water/oil emulsion and PCR reactions are performed in each droplet. This technology provides absolute quantification of PCR targets with higher efficiency and precision than conventional qPCR. Recent studies employing this technology revealed that various steps of HIV transcription including elongation, termination, and splicing are blocked in latently infected cells [43,72]. Although qPCR is a powerful and sensitive technique to detect and measure HIV RNAs, it does not differentiate signals between intact (productive) and defective RNAs, and therefore it might overestimate the size of the reservoir [5,164].

A commonly used technology to specifically measure replication-competent HIV is the quantitative viral outgrowth assay (QVOA). In this assay, stimulated PBMCs from HIV+ individuals are co-cultured with CD4+ T cells purified from HIV-seronegative individuals. Replication-competent HIV produced from HIV+ PBMCs are amplified via infecting HIV-seronegative CD4+ T cells so that viral replication can be measured by p24 ELISA or reverse transcription (RT) assays [165,166,167]. Although QVOA has been frequently used as a gold standard to measure viral reservoir bearing replication-competent HIV, there are several limitations. The assay takes weeks to obtain results, and the experiments requires a large volume blood from multiple donors. Therefore, it is time-consuming, labor-intensive and expensive. In addition, since the HIV growth depends of the condition of PBMCs, the sample-to-sample variation is often very large. More importantly, not all replication-competent viruses are stimulated and spread in this assay, resulting in an underestimation of viral reservoirs [168]. To circumvent the problem of low sensitivity, a more sensitive version of QVOA using primary cells or tissues of humanized mice [169]

## 9. Cell Line Models for HIV Latency

Using cell lines to study HIV latency and reactivation has several advantages. First, these cells are easy to maintain. HIV latently infection is easily established and single cell clones can be isolated with relative ease. High levels of DNAs, RNAs, and proteins can be ectopically expressed by lipofection or electroporation into cell lines. This facilitates genetic analysis of cellular factors and pathways involved in HIV latency and reactivation. There are several well-characterized HIV latency models commonly used to study HIV latency and test LRAs.

## 10. HIV Indicator Cells

The simplest models to study HIV transcription are highly permissive cell lines such as HeLa and HEK 293 cells which stably carry HIV LTR-driven Luc and b-Galactosidase (β-Gal), as well as cellular HIV receptors CD4 and CXCR5a. MAGI [170] and TZM-bl cells [171] are examples of such reporter cell lines. HIV can infect these cells and activates Luc genes after integration and expression of viral Tat protein. Therefore, these cells are commonly used to determine infectivity as measured by HIV titers. However, HIV LTR-driven transcription is not silenced in these cells and hence they are not an appropriate model for HIV latency. Viral Tat can still amplify HIV LTR-driven reporter gene expression by ~100 fold. Therefore, these cells are often used to study Tat-dependent transcription, but are not appropriate to study latency.

## 11. HIV Latently Infected Cell Clones

CD4+ T cells or monocyte/macrophage-originated cell lines are more physiologically related to primary cell models. There are already several different HIV latent cell clones carrying intact HIV proviruses or reporter genes under the control of HIV. Before fluorescent reporter HIV technologies were established, many HIV latent clones such as U1, A3.01, OM1.1 and J1.1. were established in the Folks and colleagues using promonocytic U937 cells, T-lymphocytic CEM and, Jurkat cells, or promyeolocytic HL60 cells chronically infected with intact HIV laboratory strains [172,173,174,175,176]. In these cells, HIV gene expression is kept at low to undetectable levels but increased by various stimulations [173]. Similarly, MOLT20-2 is isolated from lymphoblastic MOLT4 cells infected with HIV NL43 [177,178]. Early studies on HIV latency primarily used these latency models. However, measurement of HIV gene expression required labor -or cost-intensive assays such as Gag p24 ELISA, RT assay, RT-qPCR, or Western blotting. In the following sections, we will describe some of commonly used HIV latently infected cell clones (summarized in Table 2). All clones are available free to all researchers of non-profit organizations at the NIH AIDS Reagent Program (https://www.aidsreagent.org).

## 12. U1 and ACH2

U1 and ACH2 cells were among the first HIV latency models, established in the Fauci lab [174,175] by isolating U937 and A.3.01 cells chronically infected with replication-competent laboratory strains of HIV. These cells contain intact HIV proviruses without surrogate markers such as GFP or Luc. Hence, analysis of HIV gene expression from these cells requires the same methods as that of clinical samples from HIV+ individuals. Nonetheless, these cells have been widely used to study HIV latency and test LRAs. In both cells, HIV expression is blocked at the transcription level [175,176,186]. While U1 cells can be activated by ectopically expressed Tat proteins, ACH2 does not respond to Tat [186]. Genetic analysis of cDNAs and proviral DNAs revealed that U1 cells express a mutant Tat protein (H13L) which is impaired in interacting with P-TEFb [179,187]. ACH2 contains a point mutation in the TAR region that diminishes the interaction between the Tat and the TAR [188]. Therefore, while U1 cells are suitable to measure Tat-dependent and Tat-independent HIV transcription, ACH2 cells are particularly useful to study Tat-independent transcription. Recent studies revealed that HIV proviruses in both U1 and ACH2 are integrated in many different sites, 28 and 100 sites per 100,000 cells, respectively. This is in spite of the fact that both cells were originally isolated as single cell clones [189], indicating that the replication-competent HIV proviruses in these cells replicate over multiple passages. Nonetheless, the overall behavior of these cells as bulk does not seem to have changed, implying that proviral integration sites are not a main determination factor of HIV latency and reactivation in these cells.

## 13. J-Lat Series

J-Lat cells were established in the Verdin lab by infecting Jurkat cells with a VSV-G-pseudotyped replication-incompetent HIV-based lentivirus, where the HIV *nef* gene was replaced with an *egfp* gene [180]. From a pool of HIV-infected cells, five clones were extensively characterized: 6.3, 8.4, 9.2, 10.6, and 15.4 [180]. In unstimulated condition, all clones express less than 1% GFP positive cells. However, upon TNF-α stimulation, 27 to 96% GFP expression is induced [180]. Full-length HIV-based lentiviral vector, containing the intact *gag-pol* gene and the *env* gene with premature stop codon to create replication-incompetent virus, was used to make these cells Upon stimulation, J-Lat cells produce HIV VLPs into culture supernatants. Therefore, GFP expression can be measured by flow cytometry, as well as Gag p24 ELISA and the RT assay of culture supernatants, in order to analyze HIV reactivation. Simultaneously, similar HIV latent cells were established with a simpler retroviral vector containing only Tat-IRES-EGFP, Tat and EGFP are translated from the same mRNA RNA via an IRES sequence, under the control of HIV LTR [180], and several clones were isolated and characterized: A72, 82, H2, A2, and A10. These clones do not express GFP in unstimulated conditions, but after TNF-α stimulation, they highly express GFP. However, these clones do not produce VLPs since the lentiviral vector used to establish them does not encode the *gag-pol* gene [180].

All J-Lat clones are activated by most LRAs tested [14,71,109]. However, only 20–80% of LRA-stimulated cells express EGFP, even with optimal doses of LRAs [14], suggesting that a robust stimulation protocol is required for a maximum reactivation of HIV in these cells. Peterlin and colleagues demonstrated that potent TI is the cause of HIV latency in J-Lat 9.2, 8.4, and 15.4 cells, resulting in exclusive expression of HVH RNA in the unstimulated condition [46]. Further RNA-seq experiments confirmed this notion (Fujinaga, submitted). HIV provirus is integrated in highly expressed genes in these cells: *ppp5c*, *fubp*, and *uba2* for J-Lat 9.2, 8.4, and 15.4, respectively [46,189]. In J-Lat 8.4, a full-length HIV provirus is integrated into the last exons of *fubp* in the same orientation and the *nexn* gene in the opposite orientation. To reverse TI and achieve optimal HIV transcription, it is required to block on-going RNAPII transcribing the upstream or downstream host gene and induce a potent transcriptional initiation and elongation at the same time [46]. This could be fulfilled by blockage of RNAPII on the host gene and initiation of HIV transcription from viral LTR by PKC agonists combined with recruitment of active P-TEFb by P-TEFb releasers. Combinations of at least two LRAs stimulating different pathways will be required for robust HIV reactivation from latency maintained by TI [46].

## 14. 2D10

2D10 cells were established in the Karn laboratory by cloning Jurkat cells infected with an HIV-based lentiviral vector, pHR′-d2EGFP, which encodes an attenuated Tat with the same point mutation (H13L) as Tat in U1 cells [183]. Among more than 10 latent clones isolated and characterized, 2D10 cells have been most widely used to study HIV latency. 2D10 cells contain one copy of HIV provirus integrated in an exon of Selenoprotein X in the reverse orientation [183]. A main mechanism of latency in 2D10 is attenuated Tat activity, which is less capable of releasing RNAPII paused at the viral 5’ LTR by cellular NELF, although epigenetic silencing also occurs in these cells [30,183]. Although less than 1% of 2D10 cells express detectable GFP, as a surrogate marker for HIV gene expression in unstimulated culture condition, these cells respond to many LRAs robustly [97,98,183].

Since pHR′-d2EGFP encodes an EGFP fused with the d2 peptide, which promotes rapid degradation via a ubiquitin-dependent proteasome pathway, the EGFP proteins expressed by external stimuli have a very short half-life in 2D10 cells: 3.6 hrs for d2EGFP vs. >24 h for wild-type EGFP. This facilitates an efficient measurement of transcription rate. However, it also requires a special precaution for using 2D10 cell to study HIV latency particularly when protease inhibitors (PIs), including clinically approved bortezomib, are used as LRAs [116,117] since PIs can also stabilize EGFP proteins.

## 15. Other HIV Latent Clones

Other Jurkat-based latent HIV clones similar to J-Lat cells have been established in various laboratories. For example, Kutsch and colleagues isolated several HIV latent clones: A5, 3F12, 11B10, and BA5 after establishing chronic infection of HIV LTR EGFP-containing lentiviral vector to Jurkat cells. Characterization of these clones suggests that TI might be a main mechanism of viral latency [184] although subsequent studies also indicated that RNAPII-pausing by NELF might be sufficient for maintaining viral latency as suggested in 2D10 cells [185]. Similar to the J-Lat clones established in the Verdin lab, 5A8 cells were established in the Greene lab by isolating Jurkat cells chronically infected with the full-length/replication incompetent HIV EGFP virus. These cells exhibit characteristics similar to J-Lat clones [14,181,182].

## 16. HIV Latency Model vs. “Actual” HIV Latent Reservoir in HIV+ Patients

*How closely do these available HIV latent models mimic the actual latent reservoir in HIV-positive humans?* A simple answer for this question would be “probably not very much”. However, it is true that we still do not know exactly where in the human body the latent reservoirs reside, whether there are specific subsets of cells or molecular markers associated with latently infected cells, and how latency is established and maintained in these cells. Therefore, it is probably safe to say each latent model reflects at least some aspects of “real” HIV latency in vivo. Therefore, these HIV latent models are very useful to study molecular mechanisms of viral latency and search best strategies to reverse latency (“shock and kill”) or maintain latency (“block and lock”) [2,4] with the ultimate goal of achieving a functional cure of AIDS. Knowing the precise mechanism of establishment and maintenance of HIV latency in each model, choosing the right model(s), and/or using various types of models are keys to obtain highly applicable results that can lead to the development of effective therapies.

## 17. Perspective/Closing Remarks

A functional cure of AIDS requires (1) activation of replication-competent HIV from all latently infected cells plus complete elimination of these cells by cell killing mediated by host immune activity [190], virus-induced cytotoxicity [191] or killing by the LRAs themselves [192] for the “shock and kill” approach [7], or (2) complete and permanent silencing of latently infected cells bearing replication-competent HIV even after cessation of cART for the “block and lock” approach [193,194]. At this point, we have not yet developed clinically successful strategies for either approaches and a functional cure of AIDS seems far in the future. However, recent rapidly developing technologies in biomedical sciences have advanced our knowledge of how HIV persists in patients. From what we have learnt so far about the mechanisms of HIV latency, a “cocktail” of multiple compounds targeting different pathways involved in the establishment and maintenance of HIV latency will be required to optimally reactivate HIV from latently infected cells [99,195]. Taking advantage of a combination of different latency models will facilitate this approach, and provide a well-rounded picture of the mechanisms that regulate HIV latency.

## Figures and Tables

**Table 1:** Examples of LRAs.

Category	Compounds	Mechanism of Action	References
PKC agonist	Prostratin, ingenol, euphorbia kansui, bryostatin-1	Induce NFkB (canonical pathway) Increase P-TEFb proteins	[98,99,100,101,102,103,104,105,106]
SMAC mimetics	AZD5582	Induce NFkB (non-canonical pathway)	[6,107,108]
HDAC inhibitors	Panovinostat, romidepsin, vorinostat (SAHA)	Release P-TEFb from 7SK snRNP Increase H3K27Ac	[22,52,63,94,109,110,111]
BET inhibitors	JQ1, iBET	Release P-TEFb from 7SK snRNP Release Brd4 from H3K27Ac	[71,112,113,114,115]
Proteasome inhibitors	Bortezomib	Induce NFkB Increase P-TEFb proteins	[116,117]
TLR agonists	CPG 7909, Pam3CSK4, MGN1703, GS-9620	Multiple pathways?	[19,118,119,120,121,122,123]
MAPK activator	Procyanidine, cacao extract	Induce the MAPK pathway	[97,124]
DMNT1 inhibitors	5-aza-2′-deoxycytidine	Reverse epigenetic silencing, Release P-TEFb from 7SK snRNP	[48,125,126]
HKMT inhibitors	3-deazaneplanocin A, EPZ-6438, UNC-0638	Reverse polycomb-mediated transcriptional suppression	[22,127]

**Table 2:** Examples of HIV latency clones.

Cells	Host Cells	HIV Replication/VLP Production	Surrogate Marker	Integration Site	Exon /Intron	Mechanism of Latency	References
U1	U937	Yes/Yes		multiple		Attenuated Tat (H13L)	[175,179]
ACH2	A3.01	Yes/Yes		multiple		Point mutation in TAR	[174,179]
J1.1	Jurkat	Yes/Yes		multiple		ND	[176]
OM10.2	OM	Yes/Yes		ND		ND	[172]
MOLT20-2	MOLT4	Yes/Yes		ND		ND	[177,178]
JLAT8.4	Jurkat	No/Yes	EGFP	FUBP/NEXN	exon	TI	[46,180]
JLAT9.2	Jurkat	No/Yes	EGFP	PPP5C	intron	TI	[46,180]
JLAT10.6	Jurkat	No/Yes	EGFP	SEC16A		ND	[180]
JLAT15.4	Jurkat	No/Yes	EGFP	UBA2		TI	[46,180]
JLAT-A2	Jurkat	No/No	EGFP	KDM6A		ND	[180]
JLAT5A8	Jurkat	No/Yes	EGFP	MAT2a	intron	ND	[181,182]
J-Lat H2	Jurkat	No/No	EGFP	SLC25A12			[180]
2D10	Jurkat	No/No	d2EGFP *	SEPX1	exon	Attenuated Tat (H13L)	[183]
CA5	Jurkat	No/Yes	EGFP	RBM12	exon	TI/NELF-mediated RNAPII pausing?	[184,185]
BA2	Jurkat	No/Yes	EGFP	PDZD8	intron	TI/NELF-mediated RNAPII pausing?	[184,185]
11B2	Jurkat	No/Yes	EGFP	HELZ	intron	TI/NELF-mediated RNAPII pausing?	[184,185]

* shorter half-life.
